# Evaluating Spectral Signals to Identify Spectral Error

**DOI:** 10.1371/journal.pone.0146249

**Published:** 2016-01-05

**Authors:** George Bazar, Zoltan Kovacs, Roumiana Tsenkova

**Affiliations:** 1 Biomeasurement Technology Laboratory, Graduate School of Agricultural Science, Kobe University, Kobe, Japan; 2 Institute of Food and Agricultural Product Qualification, Faculty of Agricultural and Environmental Sciences, Kaposvar University, Kaposvar, Hungary; 3 Department of Physics and Control, Faculty of Food Science, Corvinus University of Budapest, Budapest, Hungary; Agricultural University of Athens, GREECE

## Abstract

Since the precision and accuracy level of a chemometric model is highly influenced by the quality of the raw spectral data, it is very important to evaluate the recorded spectra and describe the erroneous regions before qualitative and quantitative analyses or detailed band assignment. This paper provides a collection of basic spectral analytical procedures and demonstrates their applicability in detecting errors of near infrared data. Evaluation methods based on standard deviation, coefficient of variation, mean centering and smoothing techniques are presented. Applications of derivatives with various gap sizes, even below the bandpass of the spectrometer, are shown to evaluate the level of spectral errors and find their origin. The possibility for prudent measurement of the third overtone region of water is also highlighted by evaluation of a complex data recorded with various spectrometers.

## Introduction

More and more sophisticated chemometric methods are applied for finding the appropriate model to predict physical or chemical parameters of investigated samples based on their near infrared (NIR) spectra. However, users doing data processing and chemometrics often consider the spectra to be free of error, thus, underestimate the importance of checking the raw data and evaluate it with tests that can accentuate spectral regions which might have high level of noise. Rating the noise level and ascertaining the noisy intervals may become even more important when the aim is a very detailed data analysis with band assignment, or when small differences among spectra are scrutinized, thus, precise and accurate spectra are needed. Erroneous data of a certain region may highly influence the result of a spectral transformations (e.g. standard normal variate, SNV) on the entire spectrum, and can have high impact on the final precision and accuracy of an implemented prediction or classification model [1].

In general, noise means random error in the measured data. Overall error consists of random and/or biased signals carrying no useful information and may affect precision and accuracy of the results. Biased errors are often more dangerous than random noise, since the precision may be excellent, but still reflecting persistent change in the system [2]. According to Williams & Norris [2], errors are the differences between computed or measured NIR values and the true values, and can occur in both wavelengths and absorbance values. Foresaid authors declare that errors are unavoidable, thus, the best thing to do is to ensure that they are rigorously monitored and minimized, and users have to accept the limitations of each instrument. In their valuable book a complete chapter is dedicated to errors associated with the instrument, the sample, operational factors or outliers [2]. NIR technicians need to be aware of methods provided by the manufacturers to test these errors and keep them under a limit to provide creditable signal-to-noise ratio.

After all, it is very good to bear in mind the continuous existence of smaller or bigger errors. Sometimes even the term of “noise does not carry useful information” fails. Exactly, knowing the nature of noise or biased errors can help a lot in finding the real causes of the error itself, and if possible, cure these sources. It is indeed a very interesting and important procedure to find and describe errors in a given population of sample spectra. Software packages do not provide ready-to-use solutions for these tasks, so users need to evaluate and combine the possible tools. Norris and others dealt with the issue of spectral errors in several papers [2–10]. This paper provides a summary of these materials and demonstrates results achieved with the described rapid methods testing spectra. The practical approach is to seek for the origin of a persistent but not well understood signal occurred in the spectral data of some samples.

The objective of this work is to collect and describe easy to use measurement and data evaluation methods for noise detection, and decide whether a definite signal in the applied spectra recorded by a certain spectrometer is erroneous or correct.

## Materials and Methods

### Background knowledge

As a motivation for the present research, authors acquired spectra of aqueous solutions of biological samples and worked on finding the meaning of some specific wavelength ranges (unpublished results). The region between 700–800 nm considered as the 3^rd^ overtone of water (O–H) turned out to be the most interesting of all, so finding distinct pattern for different groups of samples was aimed using this spectral interval. The investigated region is at the border of visible (VIS) and NIR spectroscopy, providing signals of molecular vibrations and electron transitions. Data seemed to be accurate along the spectrum and nobody assumed that something was going wrong at any part of the data series. Later on, it was realized that the absorption around the mentioned wavelength region showed very interesting features which implied an error instead of useful signals. Thus, methods were needed for checking whether useful signals were measured, or some spectral errors were observed that accidently appeared in the water region. Accordingly, spectra of pure water and blank air were measured with the spectrometer used in the above mentioned experiment (6500-A, see below), and with other spectrometers for comparison.

### Instrumentation and software

There were three spectrometers used in the experiments detailed below. Two NIRSystems 6500 models (FOSS NIRSystems, Inc., Laurel, MD, USA) equipped with Sample Transport Module (STM) were applied (6500-A and 6500-B), recording each transmittance spectrum in the entire spectral region (400–2500 nm), at 2nm spectral steps, with 10nm nominal bandpass, as the average of 32 successive scans. Additionally, a FOSS-XDS spectrometer (FOSS NIRSystems, Inc., Höganäs, Sweden, recently Metrohm NIRSystems AG, Herisau, Switzerland) was used with Rapid Liquid Analyzer (RLA) module, and transmittance spectra were recorded in the range of 400-2500nm, at 0.5nm spectral steps, with 8nm nominal bandpass, as the average of 32 successive scans. According to the daily routine of the actual laboratory, spectrometers were not switched off, only the lamp was turned off on days when no measurement was executed.

NSAS (NIRSystems Spectral Analysis Software, version 3.53, FOSS NIRSystems, Inc., Laurel, MD, USA) was used to operate the 6500-A model and record the transmittance spectra (logT^-1^). After one hour warm-up period, the noise test of NSAS was run on each measurement day before spectrum acquisition, on the VIS (400–1100 nm) and NIR (1100–2500 nm) regions, separately. Operators using this spectrometer took reference spectrum just at the beginning of measurement series, so, no reference scan was saved between sample scans. During recording the initial reference spectrum, the whole spectral region was applied, and the internal reference standards of the spectrometer were measured, joined with a linearization, as set in NSAS settings.

In the case of 6500-B and XDS models, acquisition of transmittance spectra (logT^-1^) was performed with the VISION 3.5 software (FOSS NIRSystems, Inc., Höganäs, Sweden), and reference spectra were recorded at every sample. Successful performance tests of VISION were achieved after one hour warm-up periods before measurements. Regarding XDS, recorded spectral step of 0.5 nm was transformed to 2 nm step to get comparable data with those of the 6500 models, during derivatives with the same gap sizes. During the transformation process, absorbance values at even-numbered wavelengths were calculated by averaging every four spectral variables along the spectrum.

Microsoft Excel 2010 (Microsoft Co., Redmond, WA, USA) was used for subtractions and for the calculation of the standard deviation and coefficient of variation. The Unscrambler 9.7 (CAMO Software AS, Oslo, Norway) was used for further data processing, using the Norris Gap Derivatives [11] with various gap sizes in the derivative transformations. It must be noted, that the results may differ from those presented below when obtained with other method of derivatives (Gap-Segment Derivatives, or Savitzky-Golay Derivatives). In the authors’ opinion, the Norris Gap Derivatives method is the simplest tool to be used for the described evaluations, thus, that is in the focus of this paper. Alternatively, after adjusting the settings of the other methods of transformations, consistent results can be achieved and the same conclusions can be made.

### Experimental design

Spectra of Millipore water (conductivity 18 MΩ·cm, Direct-Q, Millipore, Molsheim, France) were recorded during repeatability and reproducibility tests, using the 6500-A model. A 1 mm open-top liquid cuvette was used for recording the transmittance spectra of each water sample. The same cuvette of water was scanned continuously in the repeatability test, at 40 seconds intervals, while in the reproducibility test water was poured out of the cuvette and refilled at each scan, and scanning interval was 2 minutes. Each newly filled cuvette of water (25±1°C) was inserted in the tempered (30°C) cuvette holder of STM for 1 minute incubation before the first scan was taken, to minimize temperature variations. Ten spectra were recorded in both tests.

Blank air was recorded with the 6500-A model, with the same settings as in case of the repeatability tests, but without any cuvette in the sample holder.

Additional experiments were performed measuring spectra of Millipore water at different temperatures between 30–60°C, using the 6500-A, 6500-B and XDS models, with the described individual settings. In each spectrometer, water filled into 1 mm liquid cuvette with top cover was heated during the experiment using thermo-regulated cuvette holder of STM (in 6500 models) or RLA module (in XDS model), and the actual temperature of the sample was recorded at the acquisition of each spectrum.

## Results and Discussion

### Evaluation of Standard Deviation and Coefficient of Variation

In a recent paper Phil Williams [10] mentions the usefulness of the standard deviation (SD) and the coefficient of variation (CV), where CV expresses the ratio of SD over the average of the spectral data. He also suggests calculating the SD for repeated scans of a sample when it is refilled each time, and for consecutive scans of an individual sample. These SD values describe the reproducibility of the measurement, and the repeatability of the instrument, respectively.

**Fig 1:**
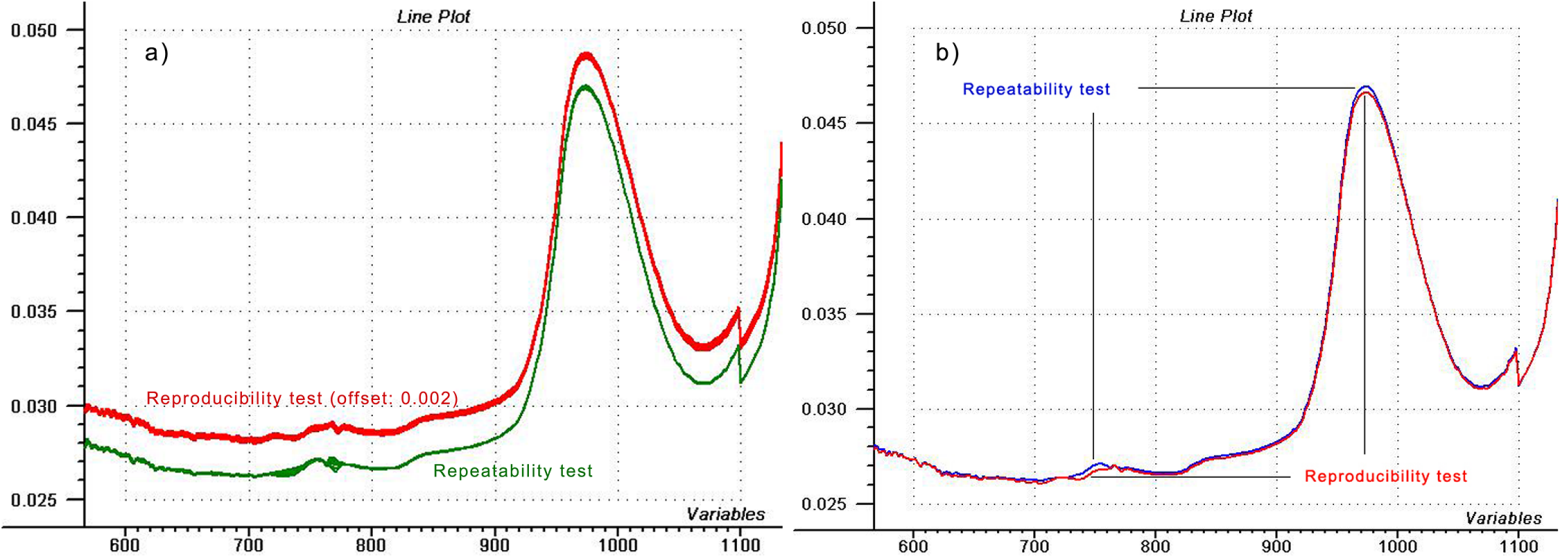
Water spectra used for performance tests. a) Plot of 10 spectra for repeatability and reproducibility tests, respectively (offset was applied for visualization), and b) the mean spectra of the two tests.

Raw and mean spectra of the reproducibility and repeatability tests performed with 6500-A spectrometer using water are shown in Fig 1. There is a definite difference around 750 nm and also at the 970 nm water peak. Water was refilled during the reproducibility test, so it did not have time to heat up, in contrary to the repeatability test where repeated scanning of the same water could cause temperature changes, thus, difference was expected.

**Fig 2:**
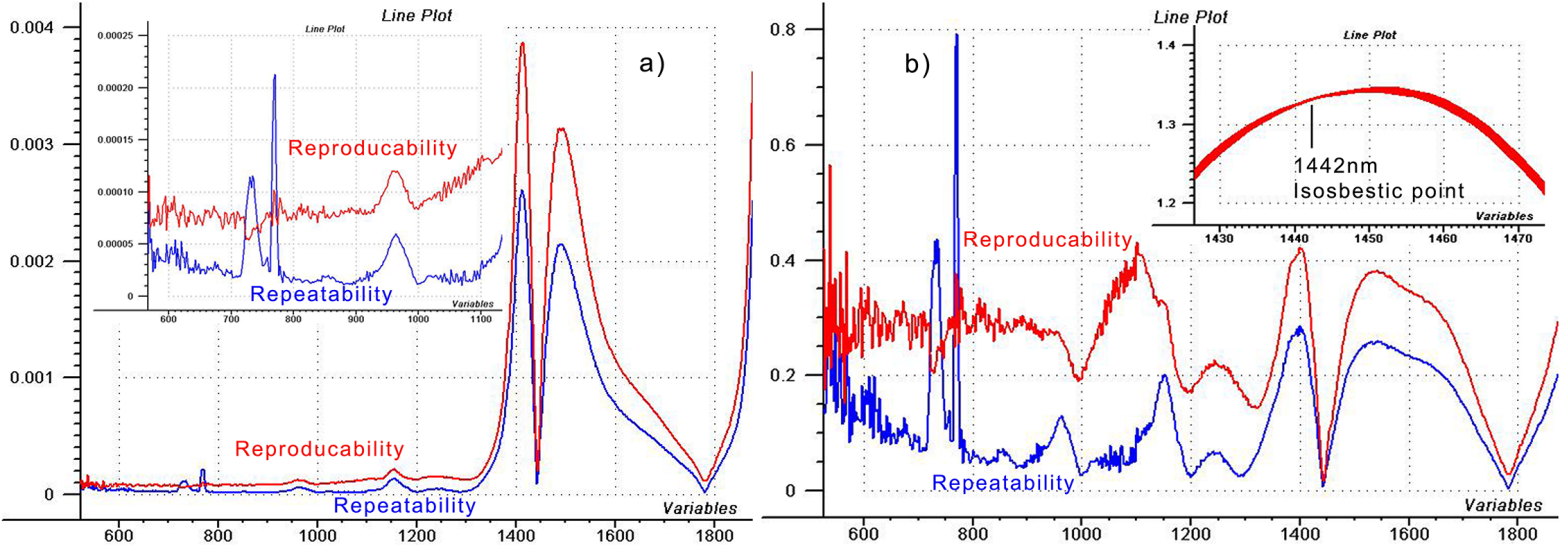
Evaluation of SD and CV of water spectra. a) SD plots and b) CV plots of the reproducibility and repeatability tests done with 10 scans, respectively.

The SD graphs of the two tests are shown in Fig 2a. It can be seen that the SD of the reproducibility test is higher all along the spectrum, because water samples were changed at each scan, placing of the cuvette might also cause some additional variations in geometry, and scanning interval was 120 seconds instead of 40 seconds, causing obvious difference in the spectral baseline (see later at air spectra). The effect of water–light interaction and continuous warming of water during consecutive scans of the repeatability measurements caused less deviation even at the 1450 nm water peak, than the fluctuations during the reproducibility test.

The very strong SD peaks of the repeatability test at 740 and 770 nm do not appear in case of the reproducibility test (Fig 2a). Since the absorption bands appear with narrow bandwidth in the short wavelength region and light interaction may cause considerable changes in water spectral pattern, we could expect that some light-caused changes were observed during the consecutive scanning of the repeatability test, which were obviously missing in case of the reproducibility test. On the other hand, if we approach the case from the prospective of spectral error, the lack of the spectral variation and the missing SD peaks of the reproducibility test do not necessarily mean that an error appears only during the acquisition of consecutive spectra. Error might appear also in case of the refilled samples, but the resulted erroneous signal could face only one direction, e.g. downwards in the spectrum, instead of the repeatability test, where it resulted positive and negative peaks randomly. Such biased error of the reproducibility test may show excellent precision (precisely wrong data), however, it reflects a persistent and erroneous change in the system [2].

Fig 2b shows the CV plots for the tests, and the graph of repeatability accentuates the problem of the 700–800 nm region. Since CV describes the relative SD, we can see that the problem of this region, where the absorbance is still very low, has much bigger impact than any other fluctuations of the spectrum at longer wavelengths where the absorbance is considerably higher.

Structural changes of water caused by light-water intercourse resulted in “blue shift” of the spectra at the water peaks during consecutive scanning [12,13]. There is a typical shape in Fig 2, at 1442 nm. The intense drop of SD and CV surrounded by big peaks indicates the position of the isosbestic point of the spectra near the 1450 nm water peak which is shifted to the lower wavelengths at increasing temperature. Obviously, SD and CV are high at this area, but nearly zero at the crossing point. The temperature shift is due to the changes in the temperature sensitive vibrational stages of free or hydrogen-bonded O–H of water, i.e. as temperature increases free O–H vibrations increase and H-bonded O–H vibrations decrease—the overlapping signals of these bonds lay beneath the 1450 nm peak [14,15].

### Subtracting the average spectrum (mean centering)

**Fig 3:**
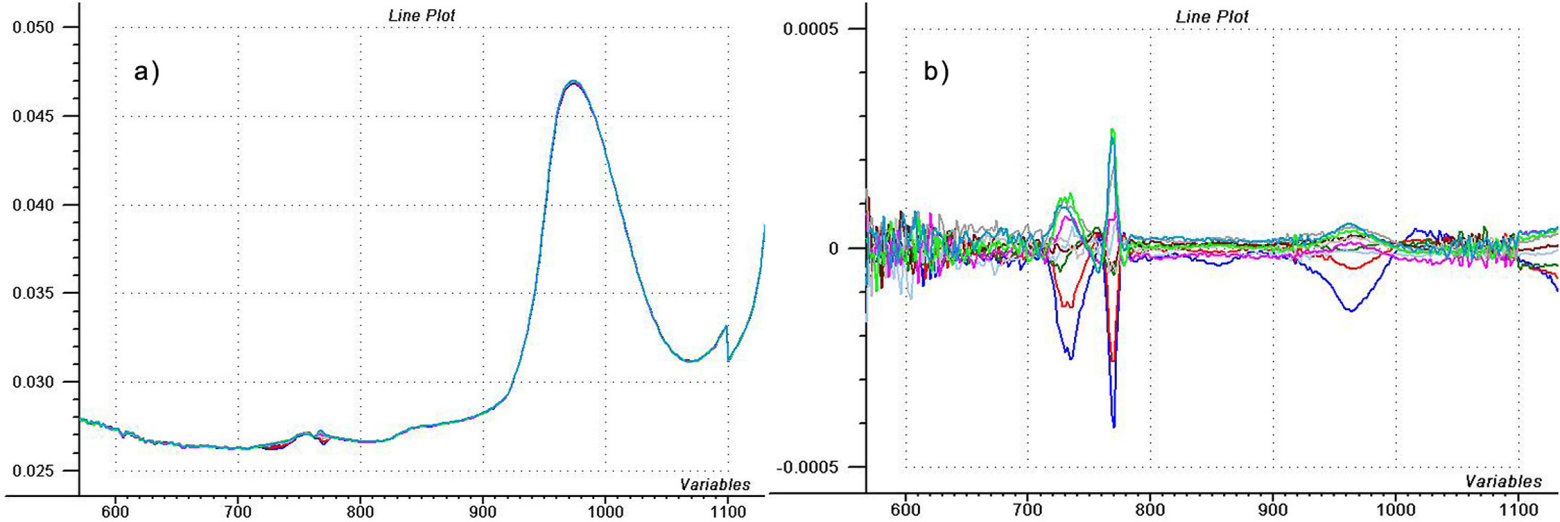
Evaluating the mean centered water spectra. a) Spectra of 10 consecutive scans of water (repeatability test) and b) the mean centered spectra of the scans.

Considering the spectra of the reproducibility test (Fig 1a), no strange things and obvious failures can be observed. But a closer look at the repeatability test shows something else (Fig 3a). These ten spectra of water were recorded consecutively, without changing the water in the cuvette. As for first sight, one may easily observe something peculiar. As already accentuated by SD and CV tests, 700–800 nm region shows great fluctuation. The slight difference in 970 nm peak might relate to the temperature change during repeated scanning. 750 nm region could also relate to this effect, but the level of the differences is extreme.

Even if the 700–800 nm spectral region seems to be false in this instrument, some questions arise: What causes this imperfection? Why don’t we see this flaw in the reproducibility test? Do we see good signals or error? To help answering this latter question, the average of a spectral population can be calculated and subtracted from all the individual ones. In the present case, raw data reflect that the result of mean centering will give very strong peaks in the 700–800 nm interval, just as Fig 3b indicates. We can also note that the peak given at 770 nm is very sharp, nearly identical with the bandpass of the spectrometer (10 nm). If this was a real signal, than the absorber should have a strong and sharp peak at this position, otherwise the represented bandwidth of the signal would be much wider just because of the relatively wide bandpass of the spectrometer. Norris [4] scrutinized this issue deeply and described the interaction among instrument bandpass, noise, absorber bandwidth and calibration error. Difference spectrum also shows deflection around 970 nm water peak (2^nd^ overtone of O–H) that might be the effect of temperature change, since difference spectrum does not reflect only the noise. What’s more, looking at the difference plots, it seems that there are some similarities between 970 nm and 700–800 nm region. Might this mean that 700–800 nm region is faultless and provides useful information on water? Further tests are necessary to answer this question.

### Subtracting the smoothed spectrum

The aforementioned tests are sensitive on errors having randomness and considered as noise. But they can hardly indicate the systematic error that has less randomness and appears with the same shape in all spectra, however, provides fake values on the investigated material.

**Fig 4:**
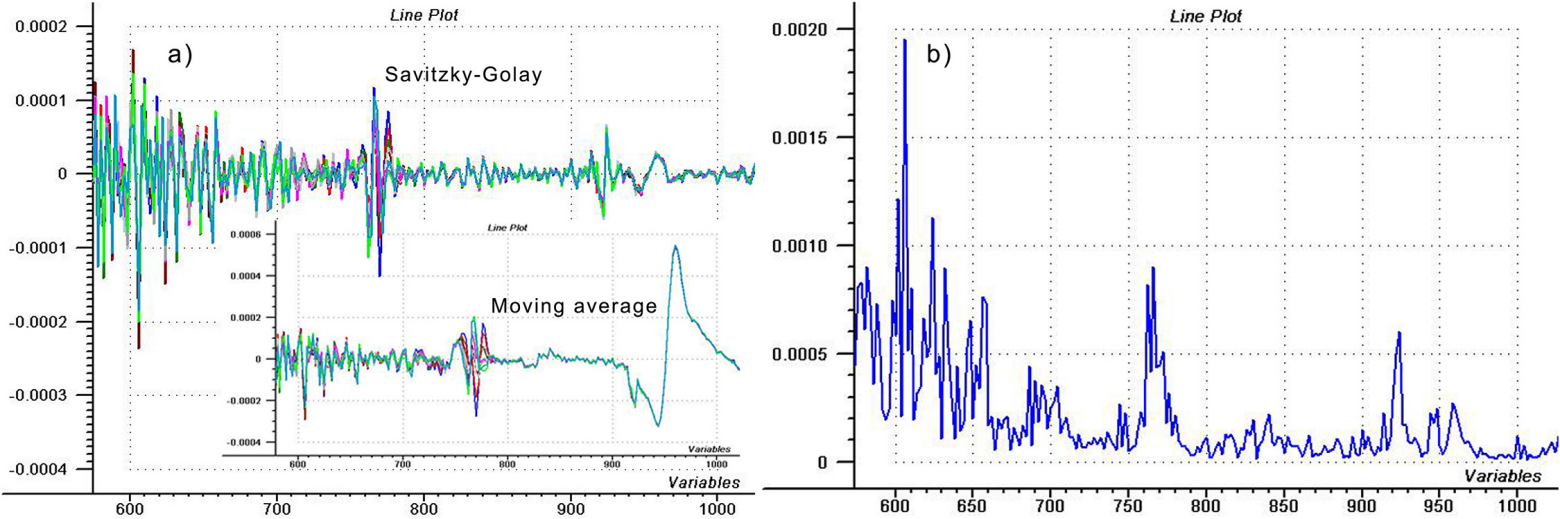
Evaluating spectra after subtraction of smoothed ones. a) Smooth-based noise spectrum of 10 consecutive water scans (repeatability test) and b) plot of cumulative noise.

Norris [3] took a closer look at the noise when he applied the Savitzky-Golay (SG) smoothing [16] for noise detection. According to this approach, smoothed spectrum is subtracted from the original one. One of the best advantages of this method is that noise can be calculated not only for a population, but for one single spectrum. Fig 4a shows the subtracted spectra of raw and SG smoothed ones, using 9 point smoothing and 2^nd^ order polynomial. The very sharp peaks indicate systematic error at the short wavelength region of each scan, decreasing to higher wavelengths, and around 750 nm the graph starts to thicken, which shows randomly appearing noise with a relatively high amplitude. It must be mentioned, that using moving average smoothing, instead of SG smoothing with polynomials, provides very different result at certain points, just as shown in the small graph in Fig 4a. If we have a wider peak, like the signals of water at the 2^nd^ and 1^st^ overtone and combination bands, and we smooth it with moving average, then lower absorbance values will appear and the peak will get even wider. So, theoretically we have to receive peaks in the smooth-based noise spectrum around the real signals, as well—however, these peaks have absolutely different shape from regular noise. We can also see the same “fake noise” in noise graph achieved with SG smoothing, but with much lower amplitude.

The possible noisiness of the different wavelength regions can be evaluated by counting the cumulative noise along the spectrum, i.e. summing up the absolute (or squared) values of noises of some scans at each wavelength. Fig 4b shows the cumulative noise counted with absolute values from the Savitzky-Golay version of Fig 4a, and the region of 770 nm provides a very intense peak, again.

The cumulative noise can be counted not only for wavelengths, but inversely, for each scan. Accordingly, the absolute (or squared) noises of all the entire wavelength range will be totalized. This measure can help selecting noisy scans, which is an important step e.g. in case of handheld or monitoring devices producing spectra loaded with considerable noise at certain conditions.

### The use of 4th derivative with varying gap sizes

Advantages of the derivatives calculated with different gap sizes were demonstrated by Norris [5] and Norris & Davies [9], using artificial data and real NIR spectra. These results showed that a small gap enhances the signal of narrow-band absorbers over that of the wide-band absorbers, and larger gaps enhance signals of the wide-band absorbers, while they can distort those of narrow-band absorbers. Karl Norris [17] recommended using derivatives of the present dataset, as well. He said, the variation in the gap of a derivative helps to identify the bandwidth of a band in the spectra, thus helps to determine what is causing the band in question: instrument, environment, sample? This is true for first, second, third, and fourth derivative, but it appears that the fourth derivative is the most efficient.

**Fig 5:**
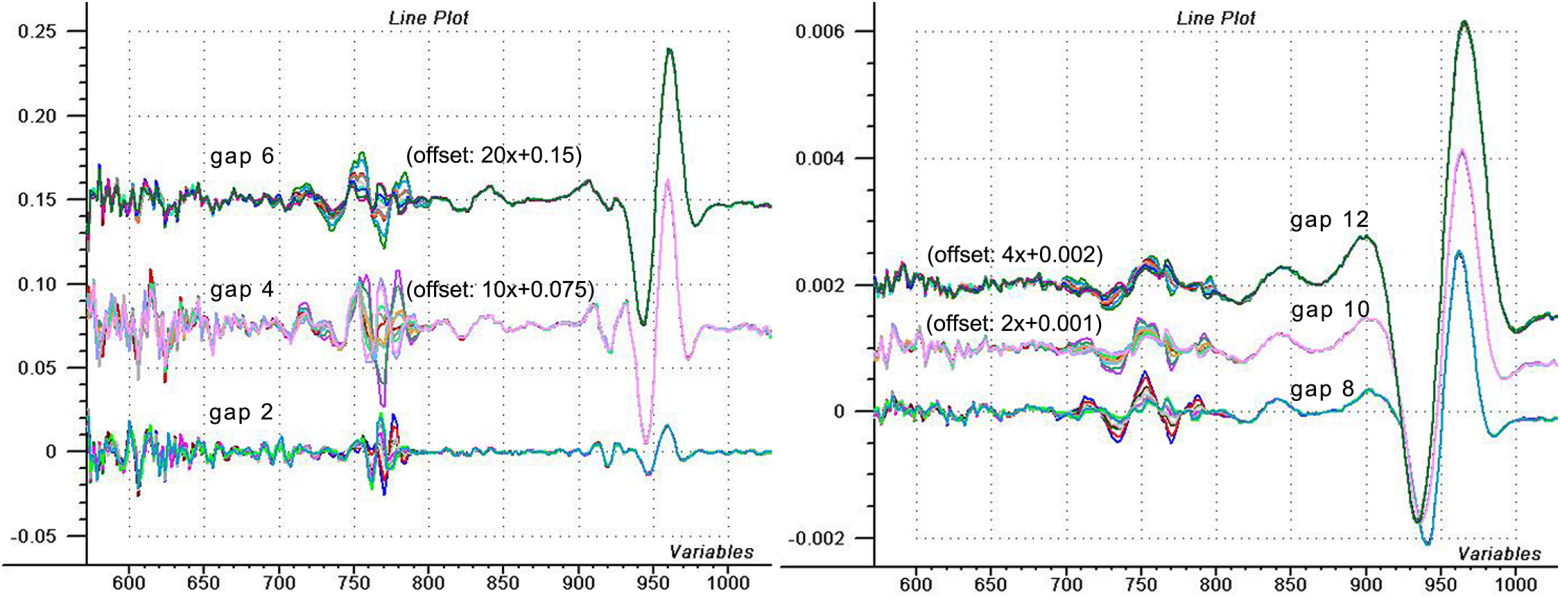
Using the 4^th^ derivatives of repeatability water spectra. Fourth derivative spectra of 10 consecutive scans of water (repeatability test) prepared with different gap sizes (different levels of offset was applied for visualization).

Fig 5 summarizes the results obtained with the 4^th^ derivatives using different gap sizes. It can be seen how 4^th^ derivative modifies the shape of peaks. Spectra are nice and sound (noiseless) at the well-defined water band at 960 nm region, in all cases. And we can see the tangled graphs of the 700–800 nm interval, where spectra show instability with the greatest variation. Spectral regions having plots as overlapping lines show stability, but this does not stand for the 700–800 nm region. This suggests that the instrument is not providing correct data at these wavelengths.

According to Karl Norris’ explanation [17], if we take a look at the 4^th^ derivative graphs prepared with bigger gaps, we can see that the spectra at 700–800 nm region do not show any similarities with the water band at 960 nm. Also, the peak at 960 nm shows no temperature change, only the variation from instrument noise. If we look at the graphs done with gaps of 2 or more points, we can see the effect referred above—i.e. with a gap of 2 points the narrow band signals in the 770 nm region are enhanced over the wider band signal at 960 nm, and with the gap of 6 or more points the opposite occurs. Derivatives with bigger gaps show the random signals of the whole 700–800 nm region very clearly. Please note that the bandwidth of an instrument noise is not affected by the 10 nm bandpass of the monochromator, because it is not a signal from the light beam. In this case the noise signals appear to have a bandwidth of about 6 nm. However, we must note that with a gap of 2 points and a data-point spacing of 2 nm our 4th derivative is wider than 4 nm. If the 4^th^ derivative is computed with a gap = 1, the bandwidth of these error signals drops to 4 nm. This definitely indicates that these bands at 770 nm are not from the sample, but from the instrument. If we measure signal narrower than the bandpass of the spectrometer, then—because of technical reasons—it must be a result of an instrument error.

**Fig 6:**
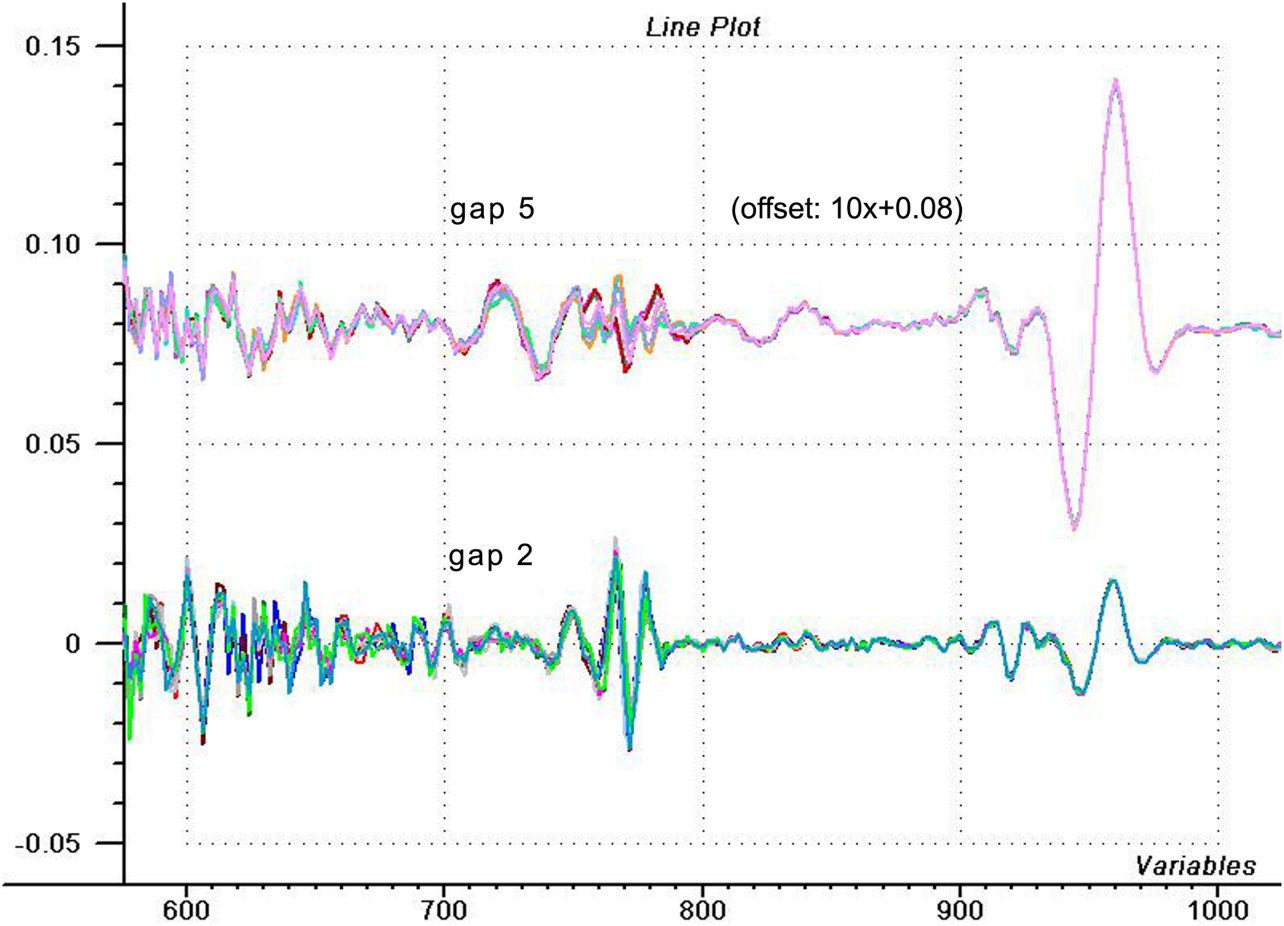
Using the 4^th^ derivatives of reproducibility water spectra. Fourth derivative spectra of 10 scans of refilled water (reproducibility test) prepared with different gap sizes (offset was applied for visualization).

And here is the answer for a former question: why don’t we see the obvious defect of the repeatability test also in case of raw spectra of the reproducibility test? Because our eyes are not always good enough to evaluate some spectra. If we check the reproducibility test by means of the 4^th^ derivatives with different gap sizes (Fig 6), then we can recognize, that our spectra are not as faultless as they seemed to be. Indeed, when small gap is used, the noise appears exactly in the same way like in the repeatability test (Fig 5)–small gap enhances the error having small bandwidth over the real absorbers having wider bandwidth (even if the absorber has very narrow bandwidth, since the recordable bandwidth is limited by the bandpass of the spectrometer) [5]. Upper spectra of Fig 6 were calculated with gap of 5 points, which actually represents the bandpass of the spectrometer. In this case, 770 nm region is not enhanced, but noisy. If we go under the recordable bandpass, and check the results of gap = 2 (Fig 6, below), we see an enhanced signal at 770 nm, while real signal of water band is suppressed. These spectra appear not so randomly as the others, but bandwidth at 770 nm region is still too narrow to be a signal coming from the sample. Talking about noise and systematic error, repeatability test shows increased random noise at the discussed region, while in case of reproducibility test this defect appears as a more systematic, biased spectral error, but it can be traced with the above described methods.

In regard to the sensitivity of different gap sizes on the bandwidths of the absorbers and the fact that the bandwidth of the same absorber varies along the spectrum (i.e. the bandwidths of higher overtones shorten with decreasing wavelength), the question appears: is it a good routine to apply the same gap size for the whole nm-spectrum during calculation of derivatives? The combination of varying gap sizes was an important theoretical value of MULR [5]. It is regrettable that there is no possibility to use it in recent software packages, since nowadays no commercial software (that authors are aware of) applies varying gap sizes within one model.

**Fig 7:**
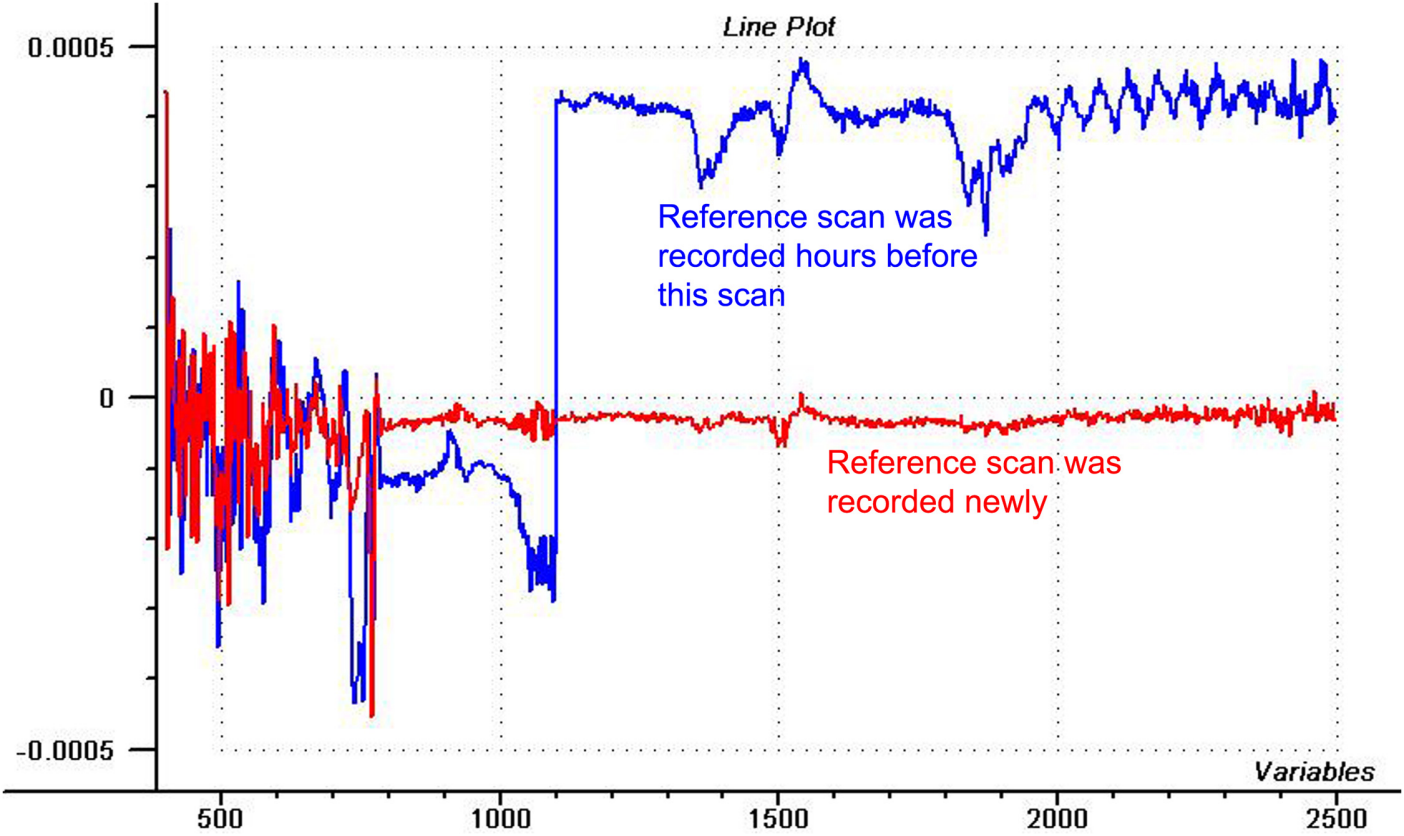
Air spectra for detecting spectral errors. Spectra of blank air recorded shortly after a reference scan or two hours later.

Not only the water spectra, but also those of blank air may help to evaluate the performance of the instrument. The mostly linear spectrum of Fig 7 was recorded directly after saving a reference scan, while the other air spectrum was measured ca. 2 hours after reference was taken. We can discover several interesting features in these spectra, especially in latter one. Spectral error at the short wavelength region of both spectra seems to be systematic, and this observation stands also for the 2000–2500 nm region of the upper spectrum. Plotting more scans showed exactly the same pattern, confirming systematic or biased error. This might be an error originated from the electronics of the monochromator that can be minimized by saving repeated reference scans. This solution stands for the huge overall baseline shift, as well. At 1500 nm we see a wave in both cases, which is a usual pattern in most of the noise spectra of 6500 models, but with repeated reference scans it can be kept below the limit of acceptance (50 μAU). The cause of this small noise at this particular place is the turning of the optical grating and Wood’s anomaly [3]. Inspecting the upper spectrum, regular water vapor peaks—originating from the changes of humidity of laboratory air circulated in the monochromator—can be registered at 1362, 1382, 1840, 1870 and 1902 nm [8]. In an optimal case, these signals—delivered by the light beam—can be minimized by repeated reference scans, as well. The level of the baseline shift (400 μAU), water vapor signal (150 μAU) and systematic error (50 μAU in NIR, 100 μAU in VIS) are far above the level of acceptance (50 μAU) a few hours after the reference scan, or already after few minutes, depending on the instrumental and environmental conditions, so, for precise measurements it is essential to record reference as often as possible. And last, but not least, we can see the extreme noise in the 700–800 nm interval, again. Fourth derivatives were calculated for these data, as well.

**Fig 8:**
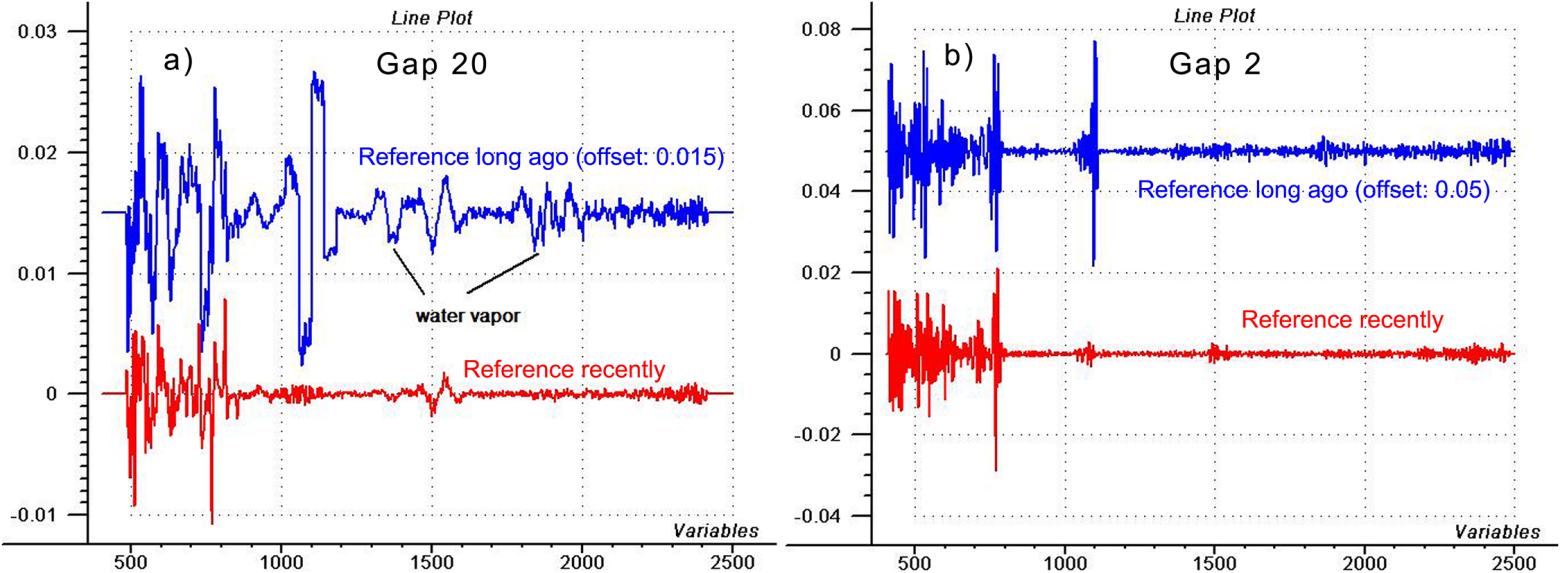
Using the 4^th^ derivatives of air spectra. Fourth derivative spectra of blank air recorded shortly after a reference scan or two hours later, prepared with different gap sizes (different levels of offset was applied for visualization).

Fig 8 shows 4^th^ derivatives of the air spectra, prepared with 20 or 2-point gap sizes. We should ignore the big signal at 1100 nm which relates to the detector switch (silica for VIS, lead sulfide for NIR region). The important thing is that the 4^th^ derivative with small gap flattens the “wide-band” signals (like water vapor) but enhances the “narrow-band” (noise) signals. With bigger gap, the wide-bands also appear.

We can get to similar conclusion as after Fig 5: since gap size of 2 points eliminated vapor peaks, but enhanced 770 nm signal, we can admit that latter must be even narrower than water vapor. Originally, vapor also has a very narrow band, but an absorber’s signal narrower than the instrument’s bandpass cannot be recorded with its real bandwidth, thus, vapor band appears much wider than it should, just because of the limitation caused by the bandpass of the monochromator (10 nm). The spectral error can be narrower than the monochromator bandpass only if the signal is coming not from the regular light beam, but e.g. from the electronics of the spectrometer. If 750 nm region represented noise of the environment (like vapor) measured by the spectrometer, it would appear as a bit wider signal.

As mentioned before, baseline shift, vapor signal and systematic noise can be minimized by taking reference scan as often, as possible. Among unstable environmental conditions, vapor might cause some difficulties and after a few sample scans it is essential to record a new reference scan. Using 6500 models or similar monochromators in extreme humid condition, there still might be some problem with appearance of humidity signal, even if we do reference scan at each sample scan. The problem might be caused by the unstable humidity of the room. 6500 monochromators circulate laboratory air and light comes through this air, collecting all of its information into the recorded spectra. Monochromator air can be so unsteady, that humidity is slightly different during reference scan and some seconds later, during the sample scan, thus, vapor signal appears in the spectra randomly, up or down. In certain sampling modules the sliding cuvette holder drawer may contain air with different humidity than the air inside the sampling module where the reference standard is measured, and this may cause unwanted vapor signals. Of course, we are talking about very small, but recognizable problems (often higher than 50–100 μAU), and we must admit, that these spectrometers were not designed for such conditions. Anyway, the solution for this type of problem in such conditions might be the application of closed monochromator, or scanning the reference and sample at the same time by dividing the light in two paths using a “double beam” spectrometer, or changing to other types of spectrometers (without monochromators) having no problem with the humidity of air.

### Disentanglement

When the investigation of the above described flaw was started, the first idea was a failure in the order sorter filter: “The model 6500 incorporates an optical filter to minimize stray light by blocking long-wavelength radiation when scanning the 400–790 nm region, and a different filter to block short-wavelength radiation for the wavelengths above 790 nm. This switching of filters during the scan generates a small noise in the recorded spectrum in this region and this noise is sensitive to instrument temperature and instrument condition.” [7]

After consultation with Karl Norris [17] and the manufacturer, the latest idea also deals with the motherboard. The mentioned 6500 model that provided the false spectral data in this particular region had a motherboard that might had problem because of its age. The old motherboard in this instrument was not able to control scanning VIS and NIR regions precisely at the same time. We tested the spectrometer by recording reference scan and sample scan only on VIS or NIR regions, separately, and the extremely high peak of 750 nm region decreased significantly. However, small deviation still remained in this region of the noise spectrum, but appeared with a lower level than the noise below 600 nm. Such level of error should not cause problem on a regular basis, but of course, it still limits the detectable difference among samples. NIR region also lost its systematic noise after reference scan was made only on that interval, so, decreasing the noise of electronics was possible with selected scanning of the VIS and NIR regions in case of this older motherboard. Of course, the problems of baseline drift and water vapor signals are not solved solely by separate VIS or NIR scanning or by a new motherboard, and often reference scan is still needed to rein these effects.

Certainly, manufacturers produce equipment to be operated as recommended—this must be kept in mind. However, if sample groups with major differences are in question, such level of noise mentioned above might not mean any problem, in general. But it can cause great troubles during finding tiny differences between samples, or during detailed band assignment.

### About the use of the discussed interval

**Fig 9:**
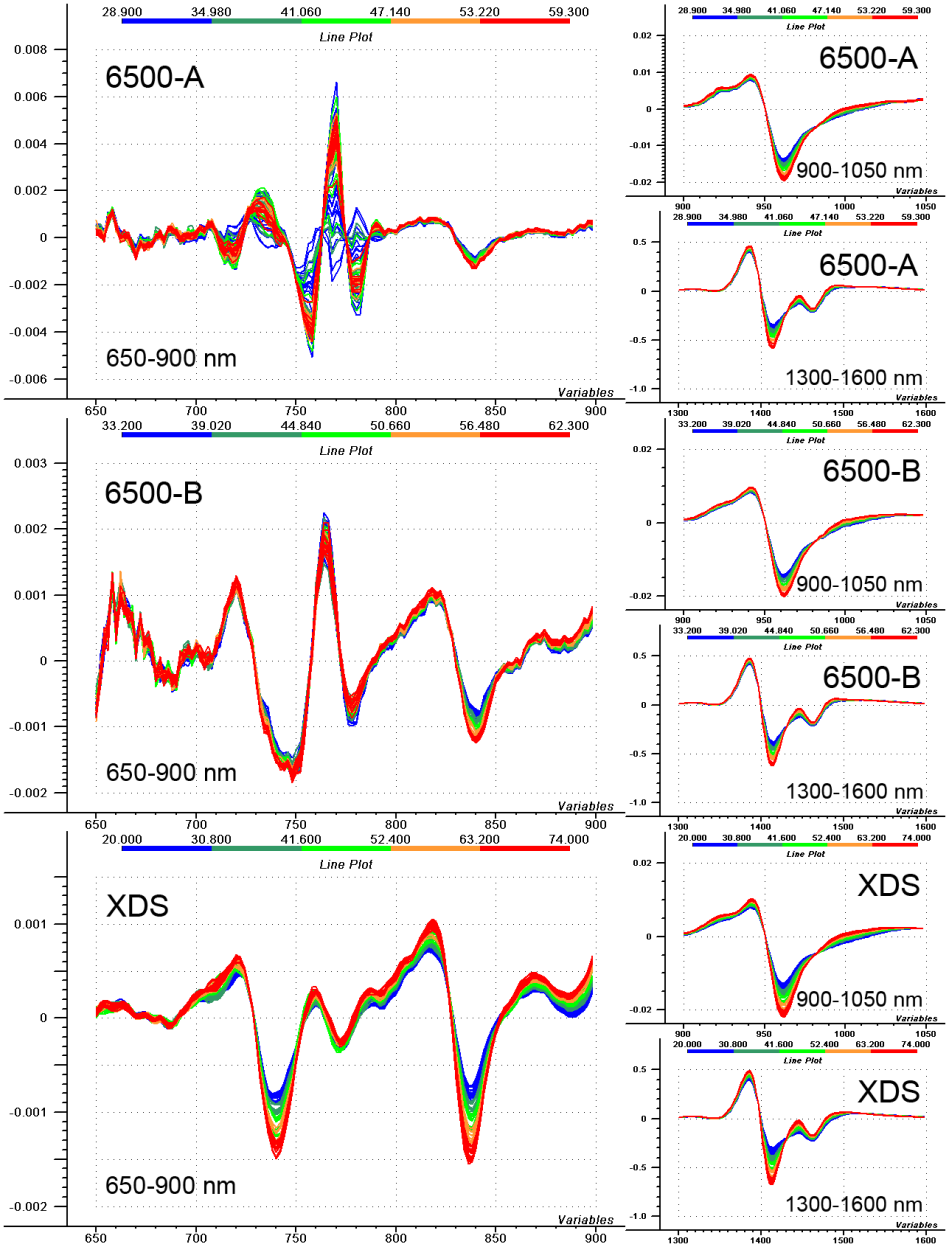
Applicability of 700–800 nm spectral interval in water measurements. Second derivative spectra (with 5-point gap) of water recorded at different temperatures (see on upper color scale), using three different spectrometers.

Authors state that the spectral interval of 700–800 nm holds useful information on water and the described imperfection applies only to a given spectrometer (6500-A) of this study. Three spectrometers with dispersive grating monochromators of the same manufacturer were tested in a temperature study, including the erroneous NIRSystems 6500 model introduced above (6500-A), another 6500 model (6500-B) and an XDS model. Fig 9 shows 2^nd^ derivative spectra of water measured at different temperatures, between 30 and 60°C, using the three spectrometers. Since the spectral step of the instruments was harmonized (as described in the Materials and Methods section), the same gap size of 5 points was applied for calculating the derivatives. The previously discussed temperature caused structural changes of water are reflected in the spectra of all instruments at the first (1300–1600 nm) and second (900–1050 nm) overtone regions of water [12–15,18].

Data obtained with the XDS model show proper water peaks also in the short wavelength region, around the third overtone of water (650–900 nm), and the quality of signals at these peaks could even be improved using cuvettes with longer optical pathlength instead of the applied 1 mm. Characteristic peaks can be found at 740 and 836 nm, showing the 3^rd^ overtone and combination bands, respectively [18], and further peaks are appearing at 772, 792 and 888 nm, but the assignment of these bands surpasses the objective of this actual paper. It is notable that the temperature dependent change at 840 nm can also be seen in both 6500 models, showing the usability of that region, just like in case of the 2^nd^ and 1^st^ overtones of water. The error of 700–800 nm interval in 6500-A is accentuated here with an increased amplitude and peak shift, while 6500-B shows very similar spectral shape as does XDS, but the changes caused by the temperature are not so nicely observable. Based on results of further inquiries and broader ranges of tests not reported here, it seems that the described spectral error of this narrow interval appears in most 6500 models because of the aforementioned order-sorter filter switching. Of course, the error is generally inconsiderable, appears with smaller amplitude and less intensive randomness (noise level below 50 μAU), thus, causing no extreme defects for example in a temperature test like the one presented here, but still, caution must always be exercised when using this particular range of those models. On the other hand, the XDS models provide very stable data (noise level around 10 μAU) with high resolution (0.5 nm spectral step with approximately 8nm nominal bandpass) even at this 700–800 nm region, just like along the whole spectrum. This test shows that the scrutinized spectral region provides useful information about water, but this information can be detected accurately only when an appropriate instrument is applied, and delicate signals of water bands are not covered by noise on the top.

All in all, the temperature test also demonstrates perfectly the problems occurred in the investigated 6500-A model. However, it is easy to admit that implementing such a circumstantial experiment is not so simple, especially not with several instruments simultaneously. On the contrary, the previously recommended short repeatability and reproducibility tests can be performed easily at any time if the perfection of a spectrometer is doubted, thus, the performance of an instrument can be evaluated quickly.

Since one series of test measurements is not enough to prove or disprove malfunction, it is necessary to repeat the mentioned rapid tests several times, iteratively, to confirm the suppositions with enough number of evidences. In the presented case, several additional rounds of tests were executed (S1 File), providing the same results, in this wise, confirming the instrument flaw and the use of the methods. Because of obvious reasons, only a short summary of the results is demonstrated in this study.

## Conclusion

The investigated spectrometer was checked before every usage, and the introduced problem was not indicated by the regularly applied noise test. The spectra looked faultless in general, and good results were obtained on them, so users of the spectrometer were more or less confident that they used a reliable instrument. Still, detailed investigation of the recorded spectra highlighted the false operation of the spectrometer. In case of basic awareness and having some doubt on our data, applying the mentioned simple “old fashion” tests, small signals appearing in our spectra can be evaluated effectively and errors can be spotted among useful signals. Knowing more about the nature of signal distortions might help to recognize the cause of these errors that might lay in sample preparation and presentation to the spectrometer, the geometry differences, the electronics, the environment, or some other factors.

## Supporting Information

S1 File Raw spectral data used for the evaluations presented in this study. Additional data sets and short “read me” files with explanations are provided to show results of repeated tests supporting the statement on the instrument flaw.
(ZIP)
